# The Role of Optimal Electron Transfer Layers for Highly Efficient Perovskite Solar Cells—A Systematic Review

**DOI:** 10.3390/mi15070859

**Published:** 2024-06-30

**Authors:** Ramkumar Vanaraj, Vajjiravel Murugesan, Balamurugan Rathinam

**Affiliations:** 1 School of Chemical Engineering, Yeungnam University, Gyeonsan 38541, Republic of Korea; ramkumar@yu.ac.kr; 2 Department of Chemistry, School of Physical and Chemical Sciences, B S Abdur Rahman Crescent Institute of Science and Technology, Chennai 600048, India; vajjiravel_m@crescent.education; 3 Department of Chemical and Materials Engineering, National Yunlin University of Science and Technology, 123 University Road, Section 3, Douliu, Yunlin 64002, Taiwan

**Keywords:** electron transport layer, titanium dioxide, perovskite solar cells, optimization of ETLs, power conversion efficiency, passivation of ETLs

## Abstract

Perovskite solar cells (PSCs), which are constructed using organic–inorganic combination resources, represent an upcoming technology that offers a competitor to silicon-based solar cells. Electron transport materials (ETMs), which are essential to PSCs, are attracting a lot of interest. In this section, we begin by discussing the development of the PSC framework, which would form the foundation for the requirements of the ETM. Because of their exceptional electronic characteristics and low manufacturing costs, perovskite solar cells (PSCs) have emerged as a promising proposal for future generations of thin-film solar energy. However, PSCs with a compact layer (CL) exhibit subpar long-term reliability and efficacy. The quality of the substrate beneath a layer of perovskite has a major impact on how quickly it grows. Therefore, there has been interest in substrate modification using electron transfer layers to create very stable and efficient PSCs. This paper examines the systemic alteration of electron transport layers (ETLs) based on electron transfer layers that are employed in PSCs. Also covered are the functions of ETLs in the creation of reliable and efficient PSCs. Achieving larger-sized particles, greater crystallization, and a more homogenous morphology within perovskite films, all of which are correlated with a more stable PSC performance, will be guided by this review when they are developed further. To increase PSCs’ sustainability and enable them to produce clean energy at levels previously unheard of, the difficulties and potential paths for future research with compact ETLs are also discussed.

## 1. Introduction

In addition to its numerous appealing photoelectronic properties and potentially low manufacturing costs, the photovoltaic industry is at present particularly interested in exploring organic–inorganic combination perovskites that feature a framework of ABX_3_ [1,2,3]. In the past few years, perovskite-based solar cells (PSCs) have exhibited an unparalleled surge in effectiveness, rising from 3.8% in 2009 to 22.7% in 2018 [4] and recently reaching 26.1% in 2023 [5]. This is the very first occasion that a novel solar cell manufacturing process has demonstrated the potential to rival existing commercially available solar cells in such a short period. Furthermore, the primary obstacles to their widespread commercialization are being progressively removed. These obstacles may be due to the instability of perovskite solar cells with respect to moisture, light etc. In order to improve their moisture resistance, the encapsulation of perovskite materials by using fluoropolymers has been reported, enabling the materials to retain 95% of their efficiency by controlling the degradation of the perovskite in the presence of moisture [6,7].

This finding suggests that long-term stability can be achieved by integrating the artificial impact of a contact sterilization strategy with the development of new, reliably stable crystals [8,9]. Achieving completely solution-based approaches, low production costs, and techniques for a streamline production process remains challenging [10,11]. Regarding the toxicological concern of lead (Pb), unleaded compounds such as MASnI_3−x_Br_x_ and MASnI_3_ have been demonstrated, which exhibited significantly poorer photovoltaic presentations in comparison to MAPbI_3_, suggesting the importance of Pb [12]. However, the tiny quantity of lead halide perovskite in these systems causes small Pb losses to have an impact on human living circumstances [13]. Consequently, there is an exciting prospect for portable and mobile energy sources because of the thorough research available on them and quick advancements in their efficiency; this should be referred to as the perovskite age rather than just a perovskite fever [14]. Electron transport materials (ETMs), which transfer electrons generated by photosynthesis from photoactive layers to the cathode, have a major impact on the efficiency of photovoltaic systems.

Based on various materials, methods, and features, there are many metal oxides that have been used as ETMs in the reported literature on perovskite solar cells. Regarding the materials, for example, titanium dioxide (TiO_2_), zinc oxide (ZnO), and tin oxide (SnO_2_) are reported as ETMs in most of the planar architecture. However, each of these metal oxides have their own advantages and disadvantages [4,15,16]. Regarding methods, spin coating or free spin coating and printing or dipping methods are employed in order to improve the coverage of the electrode as well as enhance the electron mobility [11]. Regarding features, ETLs must possess a low trap density, high light transmittance, and energy level matching, as shown in Figure 2 [11].

This review deals with the compilation of recent developments in perovskite solar cells with respect to ETMs. As discussed earlier, TiO_2_, ZnO, and SnO_2_ have commonly been used in recent research; however, the implementation of a variety of passivation strategies enhances their efficiency, stability, and processability differently. These passivation strategies, including additive/dopant engineering, thermal and solvent engineering, and interface engineering, are compiled in this review for each of these familiar ETMs. The benefits of SnO_2_ over TiO_2_ in terms of thermal processing, preparation techniques, and the nature of the materials (such as crystalline, amorphous, or nanoparticle), which are directly connected to the efficiency of the fabricated device, are discussed [17,18,19,20].

Other than the power conversion efficiency, the instability of PSCs when in contact with external stimuli such as humidity, light, or an electric field causes a severe breakdown of the perovskite crystals and plays a crucial role in their large-scale production [21,22,23,24,25]. For example, the UV-induced degradation of devices greatly affects the perovskite layer, causing carrier losses, which affect the efficiency of the device [26]. In order to overcome these issues, many attempts have been made by researchers, such as encapsulation, changing the HTL, using dopant-free HTLs, and ion migration [27,28,29,30,31,32,33,34,35]. BaSnO_3_ film doped with lanthanum (La) has also been used as an ETL in order to reduce the destruction caused by UV light, which resulted in 90% of the effectiveness being retained [36,37].

**Figure 1:**
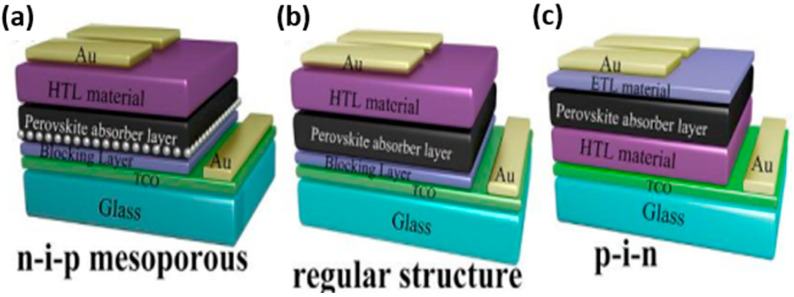
Organic–inorganic hybrid perovskites: (**a**) n-i-p mesoporous; (**b**) regular structure; and (**c**) p-i-n crystal structure [4] [reprinted with permission from *Journal of energy chemistry*, **2019**, *35*, 144–167; copyright 2024, Elsevier].

Therefore, in order to improve PSCs, understanding the structure of PSCs and the study of materials and features of ETMs are important. Regarding the structure of PSCs, perovskite solar cells have different classifications, such as being mesoporous or having a planar structure. In the case of mesoporous PSCs, these consist of ITO/hole-blocking layer/mesoporous layer/perovskite absorber/hole transport layer/metal. Mostly, mesoporous TiO_2_ or Al_2_O_3_ is used as the mesoporous layer. Initially, an efficiency of 9.7% was achieved by using mesoporous TiO_2_ with a CH_3_NH_3_PbI_3_ absorber, which was further improved to 10.9% by using a mixed-halide perovskite absorber (CH_3_NH_3_PbI_3−*x*_Cl*_x_*). By implementing different approaches such as the two-step coating method for making CH_3_NH_3_PbI_3_ [38] or solvent engineering in the preparation of CH_3_NH_3_Pb(I_1−*x*_Br*_x_*)_3_ (x = 0.1–0.15), researchers have enhanced the efficiency to 15% and 16.2%, respectively [39]. This power conversion efficiency has now reached to 22.2% through the use of printable mesoscopic perovskite solar cells (p-MPSCs) with mesoporous layers of semiconducting titanium dioxide [40]. In the case of planar structures, depending on the location of the ETLs and HTLs, regular (negative-intrinsic-positive) and inverted structures (positive-intrinsic-negative) have been classified, as shown in Figure 1.

Initially, titanium dioxide (TiO_2_) was used as the ETL in NIP structures, whereas poly(3,4-ethylene dioxythiophene) doped with poly(styrene-sulfonate) (PEDOT:PSS) was used as an HTL in PIN structures [41]. Although both architectures can currently achieve high power conversion efficiencies (PCEs) above 20–22%, NIP-type PSCs have produced significantly higher efficiencies than PIN-type architectures [42,43]. This might be the consequence of the lower open-circuit voltage (V_oc_) for PIN-type PSCs as a result of the perovskite’s inappropriate doping state close to its N-type interface, which raises the non-radiative recombination rate [44].

In the case of NIP-type PSCs, an 11.4% PCE was initially achieved for the cell structure comprising FTO/compact TiO_2_/perovskite/Spiro-OMeTAD/Au. By implementing different approaches and different deposition methods for the perovskite layer, such as the dual-source vapor deposition method [45], the sequential deposition method [46], and the doping of TiO_2_ using gold or yttrium [47], an efficiency of 19.3% was reached by 2014. However, researchers have now achieved efficiencies >20% by using different passivation strategies. For example, passivation of the interface between SnO_2_ and the perovskite by using hydroxyethylpiperazine ethane sulfonic acid achieved a PCE of 20.22% [48], and the doping of chlorine to SnO_2_ brought the PCE to 25.8% in 2021 [49].

In the case of PIN-type PSCs, PEDOT:PSS [poly(3,4-ethylenedioxythiophene):polystyrene sulfonate] and PC_61_BM or PC_71_BM ([6,6]-phenyl-C_61/71_-butyric acid methyl ester) are used as the HTL and ETL, respectively. Their ability to be prepared at low temperatures, as well as the non-requirement of HTL dopants and their compatibility with organic electronic manufacturing techniques, give p-i-n solar cells an edge over n-i-p ones. At the initial stage, by 2013, the PIN type in the sequence of ITO/PEDOT:PSS/CH_3_NH_3_PbI_3_/PC_61_BM/Al resulted in an efficiency of 3.9%. Implementing various approaches such as the one-step deposition method, the sequential deposition method, annealing, and solution processing methods led to improvements in the efficiencies of 5.2%, 7.4%, 9.8%, and 11.5%, respectively [50,51,52]. Further, the casting method or the doping of HI to perovskite solutions enabled researchers to reach PCEs of 17.7% [53] and 18.1%, respectively, by making pin-hole-free perovskite films [54]. Finally, it reached 18.9%, which was the highest during the year of 2015 [55]. However, a recent report using a polymer based on carbazole phosphonic acid (Poly-4PACz) as the HTL layer in PIN-type PSCs enhanced the efficiency to 24.4% [49].

The selection of the suitable HTL is also important towards the efficiency of PSCs. In order to reduce the recombination rate, low spatial contact is needed between the HTL and perovskite. Moreover, the highest occupied molecular orbital (HOMO) energy level in the inorganic p-type semiconductor should be at a proper position with respect to the valance band of the perovskite layer to enable proper charge transport and hole collection for obtaining a better current density [56]. Since this manuscript deals with the efficiency of PSCs with respect to the electron transport layer, details about the selection of different HTLs and the issues, challenges, and passivation strategies of HTLs are not covered in this review, and this information can be found in the literature [56,57].

Therefore, it is concluded that both of these NIP- and PIN-type architectures exhibited high efficiencies when applied with different methodologies. However, NIP types provide significantly higher efficiencies than PIN types, since NIP types provide higher V_oc_ and fill factor (FF) values. The observed discrepancy might be located at the P-type interface, where PIN-type architecture would have more difficulty extracting holes.

## 2. Systematic Literature Review

One of the most basic needs of the modern world is energy. Fossil energy resources are currently the main source of the world’s ever-rising energy demand. Fossil fuel combustion generates greenhouse gas emissions that endanger the Earth’s ecosystems by triggering global warming. To replace fossil fuels, it is therefore highly desirable to investigate alternative, carbon-free, renewable energy sources. Solar energy is a desirable electricity alternative because it is the most practical renewable energy source that might be able to meet the world’s energy requirements shortly. A solar cell is a device that directly converts solar radiation into electrical power. Solar cells are robust, dependable, and long-lasting because they do not have moving components and can operate silently and without creating any pollution [58,59,60,61]. Sustainable electrical solutions that collect environmental resources of energy (thermal, mechanical, and radiant energy) are sought after to continually power or recharge Internet-of-Things devices. Solar cells are very stable and may be produced at a low cost, among other advantageous features. Because of these qualities, solar cells are expected to be used as a long-term source of power for space probes and satellites [62].

Owing to their light absorption as well as their charge-transporting properties, silicon-based devices were focused on in earlier research [63,64]. However, their toxicity and production costs limited their bulk-scale production and thus urged for the development of a new absorber. Methylammonium lead halides (CH_3_NH_3_PbX_3_), so called perovskite materials, then emerged as a new light absorber as they can overcome the above said limitations of silicon as well as providing flexibility [65,66]. In addition to the merits of perovskite, such as the tunable band gap, high carrier mobility, high optical absorption coefficient, and longer diffusion length of carriers, it also has challenges of instability. In order to enhance the stability of PSCs, additive engineering, for example, with ionic liquid additives; compositional engineering, for example, the addition of cesium iodide (CsI); interface modification using different lead salts such as lead sulfate/lead phosphate; and different methods of dopant engineering have been carried out [67].

Regarding the ETM, especially for planar architectures, well-known metal oxides (TiO_2_, SnO_2_, and ZnO) are used; however, for inverted architectures, [6,6]-phenyl-C61-butyric acid methyl ester (PC61BM) and fullerene (C60) are commonly used as ETMs [68,69,70]. Owing to their poor filming ability and low stability, PCBMs were replaced by polymers and achieved an efficiency of 20.86% [71]. However, finding a novel ETM with appropriate energy levels, improved stability, especially towards light and humidity, and high electron mobility is still in demand. Therefore, this review mainly focuses on the development of ETMs mostly in planar architecture and the existing challenges and solutions to overcome the limitations of bulk-scale productions. The related research articles were collected and analyzed, and we compared the efficiencies of the reported PSCs with respect to the techniques or passivation strategies used.

### 2.1. Resources for the Systematic Literature Review

This systematic literature review (SLR) precisely followed the PRISMA (Preferred Reporting Items for Systematic Reviews and Meta-Analyses) criteria to methodically study the integration of transfer layers for highly efficient perovskite solar cells. Guaranteeing an organized and transparent review process was the goal. All articles published between 2018 and 2023 were included in the review, which was conducted using credible databases such as Google Scholar, IEEE Xplore, Scopus, and PubMed. Articles on processing high-efficiency perovskite solar cells’ transfer layers for optimal electron transfer were required to meet the inclusion criteria. After a rigorous selection procedure that followed the PRISMA guidelines for systematic reviews, a total of 60 articles were included. PRISMA guidelines were followed, and a thorough and methodical search strategy was used. Predefined search terms, including “Passivation of Perovskite Solar Cells”, “Surface passivation of Electron Transport Layer or Interface Layer”, “Analyzing the Perovskite solar cells with Optimal electron transfer layer”, “Role of Optimal electron transfer layer for Perovskite solar cells”, and “Perovskite solar cells with Optimal electron transfer layer”, were used to find relevant articles. Reputable databases, including Google Scholar, IEEE Xplore, Scopus, and PubMed, were searched. One thousand eight hundred articles were initially obtained from Google Scholar; two hundred ninety from IEEE Xplore; eight hundred forty from Scopus; and eighty-five from PubMed. After a thorough screening process that involved removing duplicates and determining their relevance, 280 articles were found to be eligible for additional review. PRISMA guidelines were followed in the final selection of 60 articles, guaranteeing a consistent and thorough evaluation based on the predetermined inclusion criteria.

### 2.2. Research Questions

#### 2.2.1. RQ1: Which Is the Most Efficient Electron Transport Layer for Perovskite Solar Cells?

Electron Transport Layers (ETLs) in Perovskite Solar Cells: The remarkable power conversion efficiency (PCE) and the promise of low-cost, scalable manufacture achievable with perovskite solar cells (PSCs) have attracted a lot of attention. Because they make it easier to harvest and transport photogenerated electrons, ETLs are essential to PSCs. Additionally, they aid in adjusting the interface, balancing energy levels, and reducing charge recombination inside the cell.

Optimal ETL Thickness: The effect of the ETL thickness on PSC performance has been thoroughly investigated by researchers. One noteworthy work used atomic layer deposition (ALD) to manufacture ultrathin titanium dioxide (TiO_2_) coatings as superior ETLs. The main conclusions were as follows:

Ultrathin TiO_2_ Films: Thin layers of TiO_2_ ranging in thickness from 5 to 20 nm were used in the study as ETLs.

Efficiency: By utilizing an ideal 10 nm thick TiO_2_ layer, the as-prepared PSCs on fluorine-doped tin oxide (FTO) substrates attained a noteworthy efficiency of 13.6%.

Flexible Cells: With low-temperature-processed TiO_2_ films at 80 °C, even flexible PSCs on polyethylene terephthalate (PET) substrates demonstrated an efficiency of 7.2%.

High-Performance Mechanism: Many factors were considered responsible for these cells’ success:

- The transmittance of the ultra-thin layer of TiO_2_ was increased;
- The current leakage was minimal;
- The recombination rate and resistance to charge transfer were decreased;
- The ZnO/SnO_2_ double layers outperformed all other ETLs in terms of the average power conversion efficiency, delivering 14.6% (best cell: 14.8%), which was 39% better than that of flexible cells made with SnO_2_-only ETLs in the same batch.

#### 2.2.2. RQ2: How Can a High Power Conversion Efficiency of Perovskite Solar Cells Be Achieved?

It is possible to draw the inference that PSC production must complete three key processes to reach this level of high efficiency and noticeable stability:

(1) Controlling the quality of the perovskite film;
(2) Creating the appropriate CTLs for the PSCs;
(3) Reducing flaws in the bulk and/or at the interfaces of the perovskite.

#### 2.2.3. RQ3: What Role Does the Electron Transport Layer Play in a Perovskite Solar Device?

In n-i-p architectures, the ETL is essential for producing high-performance solar cells because it inhibits recombination and encourages the transfer of photogenerated electrons from the perovskite layer to the bottom electrode.

## 3. Requirements of an Ideal Electron Transport Material

The fill factor (FF), open-circuit voltage (V_oc_), and short-circuit current density (J_sc_) have a direct correlation with the PCE. According to the concepts behind the solar power effect referred to from traditional p-i-n semiconductor designs [33], the V_oc_ is the result of the separation of both the hole and the electrons’ quasi-Fermi amounts of energy all through the whole device and is therefore impacted by the electrical energy distribution of both the perovskite lightweight film and the charge-transporting layer [72]. The light harvester’s and the device’s carrier recombination spectrum responses are reflected in J_sc_. The transport medium mobility, the film morphology, and the bulk and contact energy recombination rates in the device can all be indicators of the FF since it is directly related to charge extraction and transportation. A careful selection and architecture of the adjacent ETL are required because the current standard perovskite materials, such as FAPbI_3_ and MAPbI_3_, are moisture-sensitive, thermally unstable, and chemically sensitive due to their robust Lewis acid characteristics. Up to now, the perfect ETL should satisfy each of these specifications.

Electronic Properties: The lowest LUMO (unoccupied molecular orbital) level of the ETM ought to preferably be either somewhat lower or equivalent to that of the perovskite-based substance to facilitate electron selection. Due to the ambipolar transportation characteristic of perovskite materials, a wider band gap and a smaller maximum occupancy molecule orbital (HOMO) than those of polycrystalline active substances are needed to fulfill the electron containment and hole-blocking functionality [28]. Furthermore, there should be a decrease in the amount of material compositional disarray, which will minimize the likelihood of ETL defects in order to stop the recombination of carriers. For example, when an exceptionally ordered [6,6]-phenyl-C61-butyric acid methyl ester (PCBM) layer was placed utilizing the solvent-induced tempering process, PSCs showed an impressive rise in V_oc_ from 1.04 to 1.13 V.

A significant resistivity with electron movement larger than the polycrystalline layer of activity is also necessary to further rule out the space charge-limit effect since any charge collection at the interface would accelerate the speed of deterioration [70]. 

Features of Film Morphology: Although it stops current from flowing from small holes in the film and charge recombination at these electrode interactions, a pinhole-free dense morphological idea of the ETL is essential for highly efficient PSCs. This creates an increased shift difficulty. The ambipolar conduction property of perovskite materials is the reason for this [28]. In addition, a substantially required superior material with few flaws is needed to obtain outstanding PSCs with large V_oc_ and FF values.

Hydrophobicity and Chemical Durability: To avoid chemical reactions with the nearby epitaxial layer and anode electrodes, an ideal ETM ought to have strong chemical durability. Furthermore, because hydrophobicity keeps humidity from penetrating and interacting with the polycrystalline elements, it is crucial for ETLs in PSCs. Moreover, the interaction of chemicals that exists between the ETM and polycrystalline elements should be taken into consideration to achieve contact sterilization of the film of perovskite and lessen the interfacial carrier’s recombination brought on by defects and trap states at the electron-selective interfaces [72,73]. Additionally, because of the sensitive precipitation behavior of perovskites, selecting an ETL with an appropriate surface energy will be essential for typical n-i-p electronics to improve the kinetics of consolidation and the overall appearance of the films produced using perovskites.

While it is still very difficult to discover a single ETM that satisfies all of these requirements, several material classes and their hybrids have been researched to address PSC application requirements. Some of the crucial features of existing ETMs include the electron accessibility, the valency band maximum, and the conduction band minimum (CBM).

## 4. Electron Transport Layers in Perovskite Solar Cells

**Figure 2:**
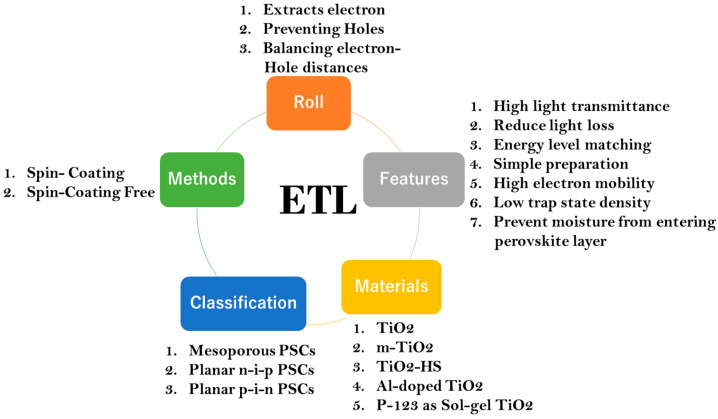
Schematic representation of the role of the ETL in perovskite solar cells.

In terms of defect states, charge transport methods, the electrical structure, thin-film manufacturing, and optoelectronic characteristics, metal oxides (MO_x_s) provide the most promising design [59]. They allow electron transit and obstruct hole transport to the corresponding electrode. Although MO_x_s reduce the voltage shunt that exists between the transparent electrode/HTL and the translucent electrode/perovskite interfaces, they have potential as materials for PSCs. A schematic representation of the role of the ETL in perovskite solar cells is given in Figure 2.

### 4.1. Titanium Dioxide (TiO_2_)

**Figure 3:**
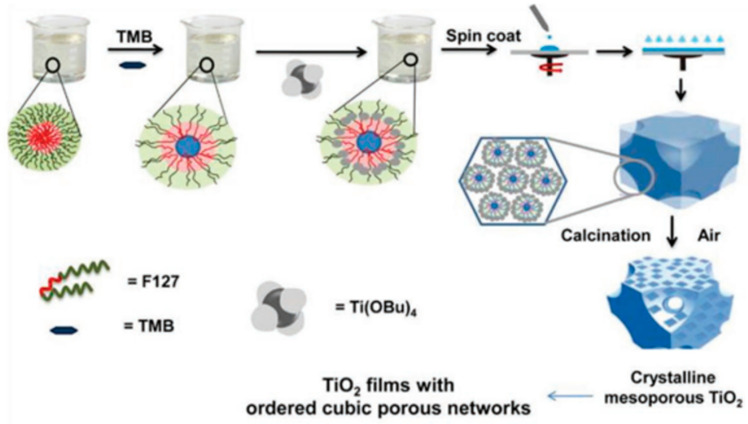
Preparation of mp-TiO_2_ film via an evaporation network [18] [reprinted with permission from *Advanced Functional Materials*, **2019**, *29*(47), 1900455; copyright 2024, Wiley].

The TiO_2_ mutations known as anatase (tetragonal), rutile (tetragonal), and brookite (orthorhombic) have been extensively employed as photocatalysts [74] and in cosmological compartments [75] due to their distinct crystalline phases and special characteristics. Due to its low cost, tunable electronic characteristics, and conductive band that closely matches that of perovskites, which facilitates electron delivery and collection, a particularly promising substance used in n-type ETLs for effective PSCs is TiO_2_. Nevertheless, there are certain disadvantages to using TiO_2_ film in PHJ PSCs: (i) TiO_2_’s poor conductivity and electron mobility make it undesirable for electron transport and collecting [76,77]. (ii) When TiO_2_ is exposed to UV light, at the substance’s interface and bordering grains, oxygen vacancies are produced. Due to this process, these vacancies act as charged traps and significantly reduce the number of carriers generated by photons [42,43]. Consequently, the contact between the TiO_2_ and polycrystalline elements causes significant instability, delaying the light-responsiveness of the resultant electronics [77]. A lot of money has been spent on changing TiO_2_ compact layers (CLs) through interfacial designs and chemical doping in order to improve PSC performance [18] (Figure 3). The surface form and properties of the TiO_2_ CL of PSCs have a significant impact on the quality of the perovskite photosensitive layer in terms of crystal size, homogeneity, and surface coverage, which in turn impacts the production of solar power [70].

#### 4.1.1. Surface Modification with TiO_2_ Nanoparticles

The change in the surface of ETLs has received a portion of consideration as a means of enhancing PSC performance and stability. The topological form of TiO_2_ films can be modified because TiO_2_ nanoparticles (NPs) have a greater specific surface area than TiO_2_ CLs. TiO_2_ NPs facilitate the effective injection of electrons and their travel, which can improve the balance of carrying charges. The TiO_2_ anatase stage is extensively used as an ETL in PSCs because it is simple to produce [78,79,80]. On the other hand, although the pure limestone stage of TiO_2_ is difficult to produce, it is the least studied phase. There is also hope for using TiO_2_’s rutile stages as an ETL for PSC purposes. Currently, in PSCs and related device structures, considering their PCEs, materials based on [6,6]-phenyl-C61-butyric acid methyl ester (PCBM) and organic materials such as self-assembling monolayers (SAMs), fullerene (C60), SnO_2_ NPs, and mp-TiO_2_ are utilized to combine with or modify TiO_2_ CL, SnO_2_, and ZnO.

#### 4.1.2. Mesoporous TiO_2_

**Figure 4:**
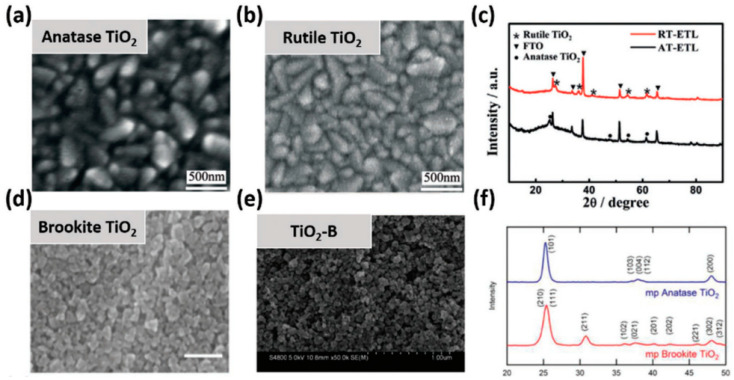
SEM photos of (**a**) anatase TiO_2_, (**b**) rutile TiO_2_, (**d**) brookite TiO_2_, and (**e**) TiO_2_-B; XRD patterns of several phases of TiO_2_ (**c**,**f**) [reprinted with permission from *Advanced Functional Materials*, **2021**, *31*(5), 2008300; copyright 2024, Wiley] [11].

The technique of fabricating mp-TiO_2_ films is often laborious and complex, involving the application of a TiO_2_ CL and then the production of mp-TiO_2_. mp-TiO_2_ necessitates a thermal sintering technique at temperatures over 500 °C to optimize its electron mobility characteristics and eliminate polymer pattern particles, in addition to changing the crystallographic state (anatase) of the aqueous oxygen sheet (Figure 3). This time-consuming, high-temperature technique limits the usage of mp-TiO_2_ in flexible PSCs produced through roll-to-roll production. Some researchers have studied how lithium-doped mp-TiO_2_ affects PSC effectiveness [34,81], and the PSCs showed better electrical properties due to the lithium-doped mp-TiO_2_ reducing the electronically charged trap states and accelerating the electron transit. The modified TiO_2_ coatings dramatically changed the electrical conductivity to improve the removal of charge and inhibit charge recombination. Furthermore, the doped TiO_2_ thin film had a major effect on the nucleation of the perovskite layer. As a result, big grains formed and accumulated to create thick films with facetted crystallites. These PSCs containing inkjet-printed mp-TiO_2_ films had a PCE of 18.29%. Large-scale applications can benefit from the dependable and scalable alternative to spin coating offered by inkjet printing technology. A PCE of 17.19% was observed in PSCs [82,83,84,85,86] that contained mp-TiO_2_ films made of 50 nm sized NPs. These films showed encouraging functions. To create nanostructure-based ETL materials for PSC applications, a great deal of work has been invested. Following this, nanopillars were employed in PSCs as ETLs. Fast carrier extraction was made possible with effective TiO_2_ CL/mp-TiO_2_ nanopillar scaffolds, which reduced the combination loss. Additional successful mp-TiO_2_-based PSCs have been reported to date. Figure 4 summarizes the energy levels of the four phases of TiO_2_ with X-ray diffraction patterns and scanning electron microscopy (SEM) illustrations [11].

In order to achieve highly efficient TiO_2_/perovskite solar cells, surface passivation has been carried out by many researchers (Table 1). For example, interfacial recombination was significantly suppressed via passivation using PMMA:PCBM in TiO_2_-based PSCs. Utilizing chlorine capping on TiO_2_ in ITO/ETL/Cs_0.05_FA_0.81_MA_0.14_PbI_2.55_Br_0.45_/HTM/metal structures resulted in a PCE of 21.40% [87]. Contact passivation with chlorine-capped TiO_2_ colloidal nanocrystals reduced the interfacial recombination and enhanced the interface binding, exhibiting an efficiency of 20.1% [88]. The doping of sodium chloride (NaCl) into a water-based TiO_2_ solution was found to improve its conductivity, energy level matching, and charge extraction in the electron transport layer (ETL) for PSCs, thus reaching an output of 23.15% [16]. In the case of carbon-based perovskite solar cells (C-PSCs), the imperfections in the bulk perovskite and at the interface between the perovskite and the electron transport layer (ETL) may lead to undesired increases in trap-state densities and non-radiative recombination, which could restrict their performance. In such cases, the passivation of TiO_2_ by using hydrogen peroxide significantly enhanced the PCE by 16.23%. H_2_O_2_-treated TiO_2_ offers a practical way to enhance the interfacial bridging between TiO_2_ and the perovskite in C-PSCs. Moreover, such passivation strategies can also enhance their long-term stability in ambient air without encapsulation [89].

In addition, doping different metals as oxides or sulfides to TiO_2_ also improved the efficiency of the devices. For example, in the case of mesoporous TiO_2_ based PSCs, Al_2_O_3_ has been used. Introducing aluminum oxide significantly suppressed the surface recombination and thus improved the efficiency [90]. In the case of sulfides, the doping of Na_2_S improved the conductivity of TiO_2_ layers. Both sodium (Na) and sulfide (S) play an important role, in which Na increases the conductivity of TiO_2_ and S alters the wettability of TiO_2_. These synergetic effects passivate the defects as well as improve the crystallinity of perovskite, and thus enhanced the efficiency to 21.25% in [91]. The doping of TiO_2_ layers using Mg had a hole-blocking effect.

Doping with Mg improved the optical transmission properties, upshifted the conduction band minimum (CBM), and downshifted the valence band maximum (VBM), with a better hole-blocking effect and a longer electron lifetime. Owing to these attributes, the resulting devices exhibited an efficiency of 12.28% [92]. Additionally, doping with indium (In) boosted the fill factor and voltage of perovskite cells. The indium-doped TiO_2_-based device consisting of Cs_0.05_(MA_0.17_FA_0.83_)_0.95_Pb(I_0.83_Br_0.17_)_3_ resulted in a 19.3% efficiency [93].

#### 4.1.3. Tin Dioxide (SnO_2_)

Owing to its favorable optoelectronic properties, such as its broad optical bandgap, elevated electron mobility, remarkable transparency in visible and near-infrared regions, suitable energetic alignment with perovskites, and effortless production of dense and transparent films through diverse methods, SnO_2_ is regarded as another feasible ETL that is commonly employed in PSCs [74,94]. Research by Miyasaka and colleagues [95] revealed that PSCs using low-temperature-processed SnO_2_ as an ETL led to a PCE of 13% with excellent stability. Another study claimed to have achieved a PCE of roughly 21% [64] by using a simple chemical bath that implanted SnO_2_ as an ETL in PSCs after processing. Surface passivation and the use of a bilayer structure are two methods for elemental doping and changing the surface. More significantly, elemental doping in SnO_2_ ETLs with different metal cations, including Li^+^ and Sb^3+^, demonstrated effective planar PSCs [59,73]. Additionally, by modifying the interface between the SnO_2_ and perovskite using a 3-aminopropyltriethoxysilane self-assembled monolayer, some researchers obtained effective PSCs with a PCE of 18% [96]. Binary alkaline halides have been employed in SnO_2_-based PSCs to apply the defect passivation approach [70]. Cesium, chlorinated Ti_3_C_2_TF, and ethylene diaminetetraacetic acid (EDTA) were used to modify SnO_2_ [97,98]. By improving the conduction band of perovskite and facilitating a smoother interface between the SnO_2_ and the perovskite, effective planar PSCs with a PCE of 21.52% were generated using EDTA [80]. Chen et al. developed PSCs with a PCE of 13.52% [34,86] by employing simple spin coating to deposit SnO_2_ onto a TiO_2_ CL to patch fractures in the TiO_2_ hole-blocking layer. Recently, stable high-performance PSCs with a PCE of 22.1% were reported wherein the TiO_2_ CL was impacted by the SnO_2_ layer [83,86]. By implementing a solution interdiffusion process, a high-quality perovskite film was fabricated with a natural drying method (without spin coating or the assistance of antisolvent, gas, or a vacuum), which improved the efficiency [99] (Table 2).

**Figure 5:**
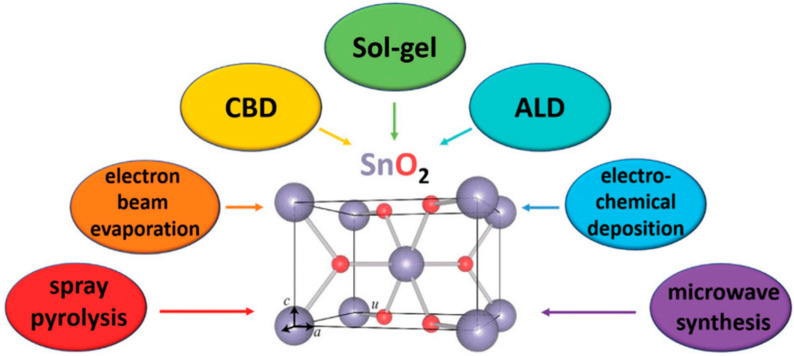
The internal structure of rutile SnO_2_ [11] [reprinted with permission from *Advanced Functional Materials*, **2021**, *31*(5), 2008300; copyright 2024, Wiley].

Mesoporous SnO_2_ ETLs were recently created using a new noncolloidal SnO_2_ precursor based on acetylacetonate. It was discovered that the halide residue in the film offers superior surface passivation to improve the hole-blocking property and is crucial to the SnO_2_’s thermal durability [11] (Figure 5).

#### 4.1.4. Zinc Oxide (ZnO)

Because of its large surface area, ease of synthesis, and low cost of production, zinc oxide (ZnO) is a great artificial semiconductor component. Moreover, ZnO has been studied the most as a CL in PSCs because of its superior optoelectronic capabilities [20]. To improve electron transmission from the perovskite layer to the ZnO ETL, the researchers added SAM across the two materials [63]. This allowed them to achieve outstandingly durable PSCs. It is possible to efficiently prevent perovskite degradation by introducing a SnO_2_ layer between the ZnO and piezoelectric layers. The PCEs of these PSCs reached as high as 12.17% with minimal repeatability. ZnO has a basic surface with a high isoelectric point (pH > 8.7), which is sufficient to remove protons from the acidic MA cation and encourage breakdown [11].

**Figure 6:**
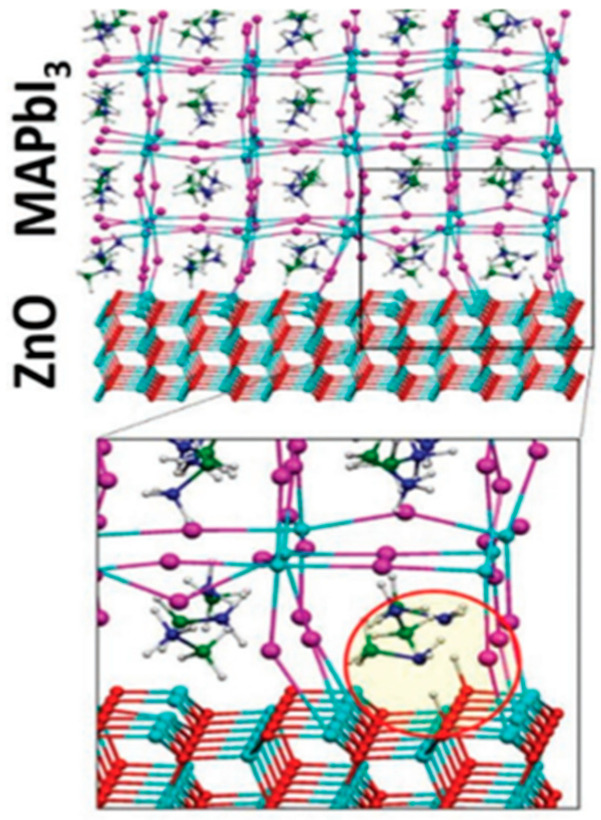
The structure of ZnO/MAPbI_3_ [11] [reprinted with permission from *Advanced Functional Materials*, **2021**, *31*(5), 2008300; copyright 2024, Wiley].

For photovoltaic (PV) devices, interface engineering in organometal halide PSCs has proven to be an effective means of improving stability and performance. Zinc oxide (ZnO) has long been recognized as a potential layer for electron transport in solar cells, and it can also be used in flexible electronics. Nevertheless, ZnO’s reactivity with the perovskite coating during the annealing process limits its use in PSCs (Figure 6). Due to the high-temperature (>450 °C) processing in producing TiO_2_-based ETLs, the fabrication of flexible devices is limited. Owing to the high electron mobility, low processing temperature, excellent optical transparency in the visible spectrum, and energy level matching with perovskites, zinc oxide (ZnO) has been considered as an alternative ETL to TiO_2_. However, achieving good efficiencies is hampered by the thermal instability of perovskite films placed directly on ZnO. Perovskite coatings on ZnO are known to break down as the post-annealing temperature rises above 70 °C. Lowering the temperature during annealing will result in partial crystallization and poor morphology of the perovskites. Therefore, the passivation of ZnO has become attractive in recent research [24,102]. For example, the surface passivation of zinc oxide using magnesium oxide and protonated ethanolamine (EA) produces highly efficient, hysteresis-free, and stable PSCs with a PCE of 21.1% [15]. MgO doping resolves the instability of the ZnO/perovskite interface. Moreover, EA promotes effective electron transport from the perovskite to the ZnO, further fully eliminating PSC hysteresis, and MgO inhibits interfacial charge recombination, thereby improving cell performance and stability [15]. However, the doping of Zinc sulfide (ZnS) on the ZnO–ZnS surface opens up a new channel for electron transport, accelerating electron transfer and lowering interfacial charge recombination. This results in a champion efficiency of 20.7% with better stability and little hysteresis (Table 3). It has been shown that ZnS improves PSC performance by acting as a passivating layer and a cascade ETL [103].

Aluminum-doped ZnO nanoparticles can improve the thermal stability of the ETL. In addition to that, PCBM (phenyl-C61-butyric acid methyl ester) can also be added to solve the problem of reduced short-circuit current density and significant photocurrent hysteresis. These modifications resulted in a PCE of 17% in [104]. Interestingly, passivation using Nb_2_O_5_ dramatically enhanced the stability of perovskite films over 20 days under ambient conditions and also exhibited an efficiency of 14.57% under simulated solar irradiation. This passivation using Nb_2_O_5_ enhanced the crystallinity of the perovskite and improved the stability of the devices [105]. A PCE of nearly 19.81% was achieved by applying interface engineering to ZnO using monolayer graphene (MLG) [61]. The introduction of MLG at the ETL/perovskite interface enhanced both the photovoltaic and carrier extraction capabilities while simultaneously shielding the perovskite layer from degradation at high temperatures, hence contributing to the device’s stability. Moreover, the efficiency was enhanced to 21% by passivating further with 3-(pentafluorophenyl)-propionamide (PFPA) [61].

In the case of ZnO-based PSCs, high stability with a PCE > 18% was achieved through the post-treatment of ZnO using ethanolamine [106]. Thus, the in situ passivation of ZnO improved the quality of the perovskite compared to that of a SnO_2_/perovskite structure.

In addition to TiO_2_, SnO_2_, and ZnO, there are some other ETLs reported in the literature [109]. Very recently, UV-inert ZnTiO_3_ was reported as an electron-selective layer in planar PSCs. ZnTiO_3_ is a semiconductor with a perovskite structure that exhibits weak photocatalysis but good chemical stability. Indium-doped tin oxide ITO/ZnTiO_3_/Cs_0.05_FA_0.81_MA_0.14_PbI_2.55_Br_0.45_/Sprio-MeOTAD/Au enhanced photostability, and displayed a stable power conversion efficiency of 19.8%. These novel ETLs offer a new family of electron-specific materials with exceptional UV stability [107].

An amorphous tungsten oxide/tin dioxide hybrid electron transport layer is also reported, which can efficiently block holes via the pinholes and cracks of the tin dioxide to indium tin oxide. This promotes charge extraction and impedes the electron–hole recombination process at the hetero-interface. Furthermore, superior electron transport is achieved in comparison to that achieved with conventional electron transport layers because of the increased mobility of amorphous tungsten oxides and the creation of a cascading energy-level sequence between the amorphous tungsten oxides and tin dioxide. A higher power conversion efficiency of 20.52% has been demonstrated by PSCs based on a hybrid ETL of SnO_2_/a-WO_3_ [108] (Table 3).

#### 4.1.5. Polymers

If utilized as an ETL scaffold, polymers can give perovskite absorbers the best possible morphologies and robust humidity resistance. However, because of their weak conductivity limits or insulating nature, mesoporous polymer scaffolds are typically employed as templates rather than ETLs in PSCs [18,19,20]. For example, a mesoporous graphene/polymer (mp-GP)/Cs_2_CO_3_ ETL can be produced at low temperatures for high-performance PSCs to enhance electron transport. The granular-like polyaniline, also known as PANI, works together with the conductive graphene network structure to perform tasks concurrently, as follows: (1) it has well-defined pores that function as quick electromagnetic frequencies; (2) it provides a permeable micro-void space for the layers of activity to infiltrate, resulting in a fully crystalline polycrystalline external; and (3) because of the chemical inactivity and packaging of the perovskite crystals, the addition of mp-GP as an ETL demonstrates increased efficiency in PSCs since the 2D version of graphite offers a solid 3D structure that protects the perovskite component from water infiltration and aggressive interface development when operating at a high frequency. Benefiting from the previously mentioned characteristics, these unencapsulated PSCs showed an impressive PCE of 13.8%, as well as exceptional chemical and thermal durability, as evidenced by a hardly perceptible drop in the PL boiling effectiveness after thirty minutes of heat annealing in air at 150 °C [86]. Polyethylene glycol was also used as a moisture-resistant component and the efficiency was recovered [110].

## 5. Future Directions and Conclusions

Perovskite solar cells with regular/planar structures exhibit efficiencies above 25%. For further development, there are many factors that need to be considered, such as improving the perovskite morphology and crystallinity (large grain size), and achieving compatibility between the ETL and perovskite absorber. In addition, the stability of the devices, low-cost fabrication, and the fabrication of flexible solar cells are other issues that remain hindrances to their widespread commercialization.

(a) Perovskite morphology: Because of the persistently high defect density in solution-processed films, effective methods for passivating these defects both in the bulk and on the surface are needed in order to achieve an efficiency of greater than 25% for commercialization. Understanding the surface morphology of both the ETL and the perovskite layer, as well as their interface, is very important before processing. Even though many attempts have been taken to improve the morphology or crystallinity of perovskites in order to minimize defects, reducing the recombination rate is still challenging. In addition to this, there is a lack of techniques or tools to qualitatively investigate or to quantify the density of perovskite defects before and after passivation. The existing steady-state PL method is limited for radiative recombination and challenges still exist for non-radiative components [111]. In order to achieve high efficiency and high-quality perovskite films with a large grain size, both an electron diffusion length that largely exceeds the optical penetration depth and high electron mobility are required. To capture more photons, additional optimizations like thickening the perovskite and adding an anti-reflection layer might be beneficial [112].
(b) Photocurrent density (V_oc_): The loss of photocurrent density (V_oc_) plays a crucial role in affecting the efficiency of perovskite solar cells. By precisely managing the perovskite preparation process, bulk impurities and structural flaws can be reduced, and non-radiative recombination losses can be avoided by controlling or engineering the layer interfaces. In this way, it is possible to achieve the full range of V_oc_ ~1.34 V for MAPbI_3_.
(c) Stability: PSC instability has been shown to be most aggressively caused by humidity because of the strong interaction between water molecules and the perovskite material. In general, ETLs have the problems of moisture sensitivity and poor film morphology. The presence of external factors such as humidity, light, heat, and an electric field, which severely damage the perovskite crystals by triggering chemical reactions or allowing ion migration to easily occur through defect sites [113]. Isolating the device from the environment, using hydrophobic back-contact materials, or encapsulating it can all be used to prevent or slow this form of degradation [114]. Encapsulation is a technique that is used to suppress charge-driven degradation. Hence, encapsulation fails to stop these molecules from penetrating, and effective mitigation techniques for charge accumulation—such as minimizing the grain-boundary defects in perovskite crystals—should be developed in order to stop irreversible degradation and enhance the material’s stability.
  A number of significant advancements have also been made in the area of long-term stability, such as the demonstration of solid-state perovskite solar cells, two-step spin-coating techniques, compositional engineering, solvent-based approaches, and the use of low-dimensional (2D, quasi-2D, and 2D/3D) perovskites [113]. In order to fix organic cations on grain boundaries and thus inhibit ion movement and ultimately significantly increase the operational stability of perovskite solar cells, a covalent bonding approach has recently been developed. Perovskites can be stabilized through ion redistributions and the release of stored charges during the nighttime via a cyclic operation that simulates an actual activity. Therefore, this covalent bond approach must be optimized by using different chemical doping methods, which may enhance the stability of the fabricated PSCs.
(d) Toxicity of Pb^2+^: Recently, lead (Pb^2+^) is still being used as the B-cation site in perovskite solar cells, even in the most advanced models. Because lead is a dangerous substance, using it could have negative effects on the environment and possibly make its way into the human food chain. One approach is the doping of Pb^2+^ by chelating it with thiol or phosphonic acid derivatives, which stop the leakage of toxic lead. Another option is the fabrication of lead-free devices.
  As a result, a lot of research has been conducted on lead-free substitute perovskite materials. Tests have been conducted on perovskite solar cells based on a variety of elements, including antimony, copper, germanium, bismuth, and others. Tin seems to be the best option because of its comparable electrical structure and ionic radius. As a result, the lead ion in the B-site can be directly replaced without causing a large phase shift. The PCE of tin-based perovskite cells is approximately 10–12%, which is substantially less than that of lead-containing perovskites. However, the drawback of tin is that it undergoes oxidation from Sn^2+^ to Sn^4+^. Therefore, doping with suitable elements or chemicals need to be optimized.
(e) Commercialization: There are still some major issues stopping the large-scale commercialization of perovskite solar cells. The current manufacturing techniques used in lab-scale projects are not suitable for large-scale production. This is being addressed with a search for techniques that are compatible with roll-to-roll processing, allowing high throughput.

In conclusion, researchers and scientists are developing next-generation PSCs with enhanced PCE and long-term stability in an effort to solve these difficulties. Furthermore, to completely unlock the high inherent electrical quality that perovskites offer, appropriate passivation procedures, including dopant engineering, solvent engineering, interface engineering, and heat engineering, must be developed. Perovskite has the potential to surpass other PV technologies in the future with the help of methodical collaboration between a variety of scientific, engineering, and entrepreneurial sectors.

## Figures and Tables

**Table 1:** Descriptions of different surface alterations in TiO_2_-based devices and their PCEs.

Passivation of TiO_2_	Device Structure	PCE (%)	Ref.
Cl-capped TiO_2_	Cs_0.05_FA_0.81_MA_0.14_PbI_2.55_Br_0.45_	21.40	[87]
PMMA:PCBM on TiO_2_	Cs_0.07_Rb_0.03_FA_0.765_MA_0.135_PbI_2.55_Br_0.45_	20.40	[88]
NaCl-doped TiO_2_	ITO/TiO_2_:NaCl/perovskite/Spiro-OMeTAD/Au	23.15	[16]
H_2_O_2_-TiO_2_	ITO/H_2_O_2_-TiO_2_/Cs_0.17_FA_0.83_Pb(I_0.83_Br_0.17_)_3_/Spiro-OMeTAD/carbon	16.23	[89]
Al_2_O_3_-TiO_2_	ITO/c-TiO_2_/A-Al_2_O_3_/mp-TiO_2_/FAPbI_3_ -MAPbBr_3_/PTAA/Au	17.92	[90]
Na_2_S-doped compact TiO_2_	FTO/Na_2_S-doped TiO_2_/PVK/BCP/Ag	21.25	[91]
Mg-doped TiO_2_	ITO/Mg-doped TiO_2_/TiO_2_:CH_3_NH_3_PbI_3_/Spiro-OMeTAD/Au	12.28	[92]
Indium-doped TiO_2_	ITO/In:TiO_2_/Cs_0.05_(MA_0.17_FA_0.83_)_0.95_Pb(I_0.83_Br_0.17_)_3_/Spiro-OMeTAD/Au	19.30	[93]

**Table 2:** Descriptions of different surface alterations/device architectures and their PCEs.

Surface Modification	Device Structure	PCE (%)	Ref.
High-efficiency large-area pin perovskite solar cells	CsPbI_0.05_[(FAPbI_3_)_0.89_(MAPbBr_3_)_0.11_	19.83	[75]
Inorganic hole transporting materials	CuSCN and Cu:NiO_x_ HTMs	20.00	[76]
Polyelectrolyte-doped SnO_2_	PSCs based on SnO_2_:PEIE ETLs	20.61	[68]
Cl-incorporated tri-cation	Cs_x_FA_0.2_MA_0.8−x_Pb(I_1−y_Cl_y_)_3_	20.31	[69]
SnO_2_ electron transport layers	Nd^3+^ ion with SnO_2_	20.92	[71]
Hydrothermally treated SnO_2_	PSCs based on SnO_2_ ETLs	18.10	[81]
TiO_2_ ETL for highly efficient and hysteresis-free planar perovskite solar cells	SnO_2_/c-TiO_2_ ETL	21.40	[85]
Metal oxide nanoparticle charge extraction layers	Triple-cation perovskite cells	18.60	[86]
Aqueous-solution-processed 2D TiS_2_ as an electron transport layer	Planar Pero-SCs	18.90	[100]
Perovskite photovoltaic modules achieved via cesium doping	MAPbI_3_-based perovskite modules	18.26	[10]
SnO_2_ modified with RbCl and potassium polyacrylate (K-PAM)	ITO/SnO_2_/(FAPbI_3_)_1−x_ (MAPbBr_3_)_x_	24.07	[101]

**Table 3:** Descriptions of different surface alterations in ZnO-based and other ETL-based devices and their PCEs.

Passivation of TiO_2_	Device Structure	PCE (%)	Ref.
MgO-protonated ethanolamine with ZnO	ITO/MgO:ZnO(CsFAMA)Pb(BrI)_3_/HTM/Au	21.08	[15]
ZnO-ZnS	ITO/ZnO-ZnS/CsFAMA/Spiro-OMeTAD/Au	20.70	[103]
Aluminum-doped zinc oxide (AZO)	ITO/Al-ZnO/PCBM/perovskite/Spiro-OMeTAD/Mo_x_-Al	17.00	[104]
Nb_2_O_5_-ZnO	ITO/ZnO-Nb_2_O_5_/perovskite/Spiro-OMeTAD/Au	14.58	[105]
Graphene/ZnO	ITO/graphene-ZnO/perovskite/Spiro-OMeTAD/Au	19.81	[61]
Ethanolamine ZnO nanoparticles	ITO/ethanolamine-passivated ZnO/FA_0.9_Cs_0.1_PbI_3_ Spiro-OMeTAD/Au	18.00	[106]
ZnTiO_3_	(ITO)/ZnTiO_3_/Cs_0.05_FA_0.81_MA_0.14_PbI_2.55_Br_0.45_/Sprio-MeOTAD/Au	19.8	[107]
a-WO_x_/SnO_2_ hybrid	ITO/WO_x_-SnO_2_/perovskite/Spiro-OMeTAD/Ag	20.52	[108]

## Data Availability

No new data were created or analyzed in this study. Data sharing is not applicable to this article.
